# Japanese public health nurses classified based on empathy and secondary traumatic stress: variable-centered and person-centered approaches

**DOI:** 10.1186/s12888-023-05198-6

**Published:** 2023-10-02

**Authors:** Masato Kitano, Kotaro Shoji, Ikumi Nakaita, Shinya Sano, Shoichi Tachibana, Jun Shigemura, Hisateru Tachimori, Norihito Noguchi, Fumiko Waki, Naoki Edo, Minori Koga, Hiroyuki Toda, Aihide Yoshino, Masanori Nagamine

**Affiliations:** 1 Division of Behavioral Science, National Defense Medical College Research Institute, 3-2 Namiki, Tokorozawa City, Saitama 359-8513 Japan; 2 University of Human Environments, Okazaki, Aichi Japan; 3 Faculty of Nursing, Musashino University, Tokyo, Japan; 4 Department of Psychology, National Defense Medical College, Saitama, Japan; 5 Division of Environmental Medicine, National Defense Medical College Research Institute, Saitama, Japan; 6 Department of Psychiatry, National Defense Medical College, Saitama, Japan; 7 Endowed Course for Health System Innovation, Keio University School of Medicine, Tokyo, Japan; 8 Department of Information Medicine, National Institute of Neuroscience, National Center of Neurology and Psychiatry, Tokyo, Japan; 9 Department of Nursing, National Defense Medical College, Saitama, Japan

**Keywords:** Empathy, Secondary traumatic stress, Public health nurse, Person-centered approach

## Abstract

**Background:**

Healthcare providers frequently help traumatized people and are regularly exposed to indirect trauma from their work, resulting in negative psychological responses, such as secondary traumatic stress. Empathy has been associated with patient’s quality of care and secondary traumatic stress among healthcare providers. However, the relationship between dispositional empathy and secondary traumatic stress has not been fully elucidated. This study used person- and variable-centered approaches to explore the nature of this relationship.

**Methods:**

A total of 1,006 Japanese public health nurses working in the Tohoku region and Saitama prefecture completed questionnaires that included scales assessing dispositional empathy, secondary traumatic stress, and burnout. First, we examined predictors of secondary traumatic stress using multiple linear regression analysis. Then, we conducted a latent profile analysis to classify participants into unique groups based on four subscales of dispositional empathy (i.e., empathic concern, perspective taking, personal distress, fantasy) and secondary traumatic stress. Finally, we compared the mean values of the study variables across these groups.

**Results:**

The multiple regression indicated that in those working in Saitama prefecture, lifetime traumatic experiences, work-related distress, and personal distress were positively related to secondary traumatic stress, but perceived support was negatively related to secondary traumatic stress. Latent profile analysis extracted four unique subgroups. Group 1 displayed the highest secondary traumatic stress levels. Group 2 was characterized by the highest level of empathic concern, personal distress, and fantasy and the lowest perspective taking. Group 3 had a moderate secondary traumatic stress level. Group 4 had the lowest secondary traumatic stress and personal distress scores. In these four groups, the burnout scale (exhaustion, cynicism, and professional efficacy) showed a pattern similar to the secondary traumatic stress scale.

**Conclusions:**

Our person-centered approach showed that this sample of public health nurses could be classified into four unique groups based on their empathy and secondary traumatic stress scores. Although this group of public health nurses was not large, one group displayed high personal distress levels and high secondary traumatic stress levels. Further research is needed to determine effective interventions for this group.

**Supplementary Information:**

The online version contains supplementary material available at 10.1186/s12888-023-05198-6.

## Background

Healthcare professionals are frequently exposed to indirect trauma by constantly assisting those traumatized, which may have adverse psychological effects including secondary traumatic stress (STS) [1]. STS refers to post-traumatic stress disorder-like stress reactions (i.e., intrusive thoughts about the trauma, avoiding trauma triggers, and physiological arousal) that result from indirect traumatic exposure [1]. Notably, 90.3% of Japanese nurses have experienced indirect trauma through their work [2]. The prevalence of STS is 15.2% among social workers [3], 19% among substance abuse counselors [4], and 32.8% among emergency nurses [5]. Furthermore, social workers who worked with disaster survivors after the 2011 Great East Japan Earthquake had greater STS than those who did not work with the survivors [6]. Moreover, Japanese disaster workers exposed to corpses or increased exposure to earthquake survivors had greater STS symptoms after the 2011 Great East Japan Earthquake than those without these experiences [7].

Researchers and healthcare providers have viewed empathy as a characteristic of healthcare providers associated with patient care quality. Empathy is the capacity to identify and understand others’ responses to their experiences when they are observed and is a core characteristic of social animals, including humans [8, 9]. It helps maintain and facilitate social relationships, understand social behaviors, and encourage cooperation.

The multidimensional model of empathy has posited that dispositional empathy comprises both emotional and cognitive components [8, 10], with personal distress as an emotional component that entails experiencing distress and anxiety when exposed to another’s negative affect [8]. The emotional component of empathy facilitates its cognitive component that helps an observer speculate on another’s intentions, emotions, beliefs, and motivations [11]. The cognitive component of empathy, or perspective taking, refers to spontaneous attempts to adopt another’s perspective and understand what they feel, think, and believe [8, 12].

Physicians’ expressions of empathy are associated with increased patient satisfaction, trust, autonomy support, and knowledge [13]. Moreover, nurses’ empathy has a robust inverse association with patients’ distress [14]. Thus, healthcare providers’ empathy can lead to better patient treatment outcomes.

However, dispositional empathy has also been related to STS [15]. A study of 7,584 physicians in Argentina reported that higher empathic concern and personal distress were associated with elevated STS [16]. Among Canadian neonatal and pediatric intensive care unit nurses, STS has a moderate and positive relationship with empathic concern, perspective taking, and personal distress [15]. Conversely, a study among Jordanian emergency room nurses demonstrated a weak negative association between empathy and STS [17]. These inconsistencies could result from limited sample sizes, differences in measures, cultural differences, and nurse specialties. Similarly, previous studies have reported mixed results on the relationship between dispositional empathy and burnout [14–16, 18].

Immediately after the Great East Japan Earthquake in 2011, public health nurses were ones of front-line workers who potentially experienced extreme distress by helping others. During the aftermath of the earthquake, the roles of public health nurses included visiting shelters for health and hygiene management for the evacuees, visiting homes of people who needed support, and supporting the restart of health services [19]. As they had many direct interactions with earthquake victims, they were expected to have relatively high STS levels. The present study examined the association between dispositional empathy and STS among Japan’s relatively large sample of public health nurses.

## Person-centered approach

Although a person-centered approach is used less frequently than a variable-centered approach, its popularity has increased [20]. Such an approach attempts to identify the optimal number of subpopulations within a sample using a set of variables of interest. It investigates the relationships between the subpopulations with a set of variables, such as demographic variables. For example, one study used a latent profile analysis to identify four subgroups among mental health clinicians based on different empathy components [21]. Thus, a person-centered approach can help identify crucial subpopulations based on dispositional empathy. Previous studies demonstrated that dispositional empathy and STS have complex relationships [15–17]. These previously mixed findings might indicate that subpopulations with high STS and specific empathic traits exist. Identifying the subpopulations using these variables can be essential for creating a psychoeducational intervention that targets empathy to reduce STS. However, to the best of our knowledge, no study has examined subpopulations of Japanese public health nurses based on dispositional empathy and STS, and the differences in work-related outcomes, such as burnout between these subpopulations. We explored if Japanese public health nurses who regularly worked with people with traumatic experiences could be classified into meaningful subpopulations based on dispositional empathy and STS components.

## Methods

### Participants

Public health nurses (*n* = 2,085) working in the Tohoku region of Japan (Fukushima, Miyagi, and Iwate prefectures), where the 2011 Great East Japan Earthquake occurred, were recruited as potential participants. We also recruited 1,019 public health nurses working in Saitama prefecture, a neighboring prefecture of Tokyo. Of these 3,104 potential participants, we received 1,259 survey packets, representing a 40.6% return rate.

**Fig. 1:**
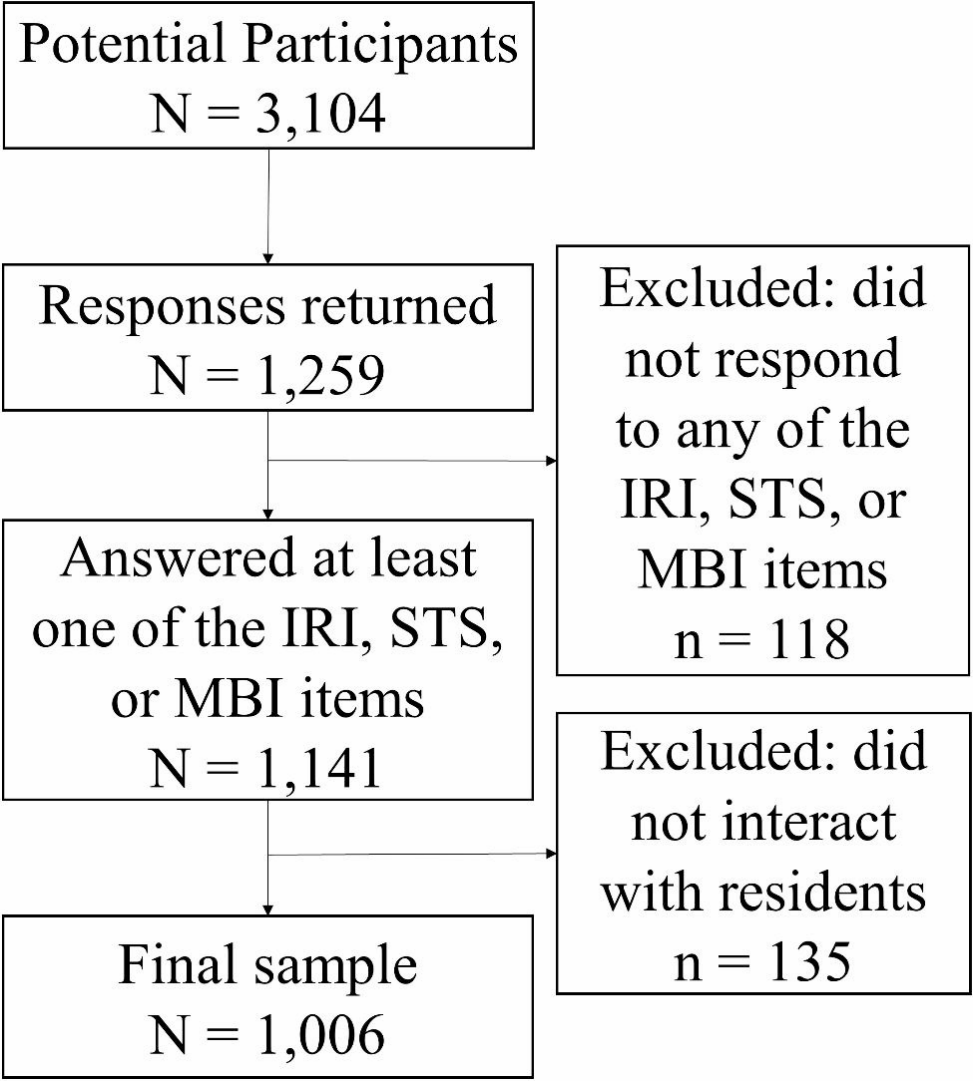
Flowchart of the Participant Inclusion Process

In the Tohoku sample, 889 public health nurses returned their survey. Among them, 99 did not fill out any of the items of the Interpersonal Reactivity Index-Japanese version (IRI-J), Secondary Traumatic Stress Scale-Japanese version (STSS-J), or Maslach Burnout Inventory-Japanese version (MBI-J), resulting in 790 valid responses from participants. In the Saitama sample, 370 public health nurses returned the survey; however, 19 did not complete any items on the IRI-J, STSS-J, or MBI-J, resulting in 351 valid responses. As we investigated the effect of indirect trauma exposure through interaction with clients, 96 and 58 responding public health nurses, who lacked direct interaction with clients as a part of their job were excluded from the Tohoku and Saitama samples, respectively. The final sample included 1,006 (Tohoku *n* = 694; Saitama *n* = 312) public health nurses. A flow chart of participant recruitment and inclusion is shown in Fig. 1.

### Measures

**Dispositional empathy**

Dispositional empathy was measured using the IRI-J [22]. The 28-item IRI-J assesses empathic concern, personal distress, perspective taking, and fantasy on a 5-point scale from 1 (*does not describe me well*) to 5 (*describes me very well*). The fantasy subscale measures the tendency to be deeply involved in, and associate their feelings with, fictitious characters in movies, books, or plays [8, 22], such as “I often have tender, concerned feelings for people less fortunate than me,” “In emergencies, I feel apprehensive and ill-at-ease,” “I sometimes find it difficult to see things from the ‘other guy’s’ perspective,” and “I daydream and fantasize, with some regularity, about things that might happen to me.” Internal consistency coefficients (α) for this study were .65 for the empathic concern subscale, .61 for the perspective taking subscale, .61 for the personal distress subscale, and .76 for the fantasy subscale.

**Secondary traumatic stress**

STS was assessed using the STSS-J [23]. The original STSS is a 17-item self-rated measure that assesses the frequency of STS symptoms (reexperiencing, avoidance, and arousal) over the last seven days [1]. Respondents evaluated the frequency of each symptom concerning their work with clients exposed to trauma using a 5-point scale from 1 (*never*) to 5 (*very often*). Sample items included “I felt emotionally numb,” “I wanted to avoid working with some clients,” and “I was easily annoyed.” The internal consistency coefficient for the present study was 0.94. The STSS-J is available upon request.

**Burnout**

Burnout was assessed using the MBI-J [24, 25]. The 16-item MBI has three subscales that measure exhaustion, cynicism, and professional efficacy rated on a 7-point scale from 1 (*never*) to 7 (*always*). Exhaustion refers to the feeling of overextending and depleting one’s emotional and physical resources. Cynicism refers to negative, callous, or excessively detached responses to various aspects of a job. Professional efficacy refers to feelings of competence, achievement, and productivity at work [26]. Sample items included “I have negative thoughts about my job,” “I felt I am achieving less than I should,” and “I am frustrated with parts of my job.” Internal consistency coefficients for this study were 0.91, 0.85, and 0.88 for exhaustion, cynicism, and professional efficacy, respectively.

**Perceived support**

We collected data on perceived support for work-related issues using two items designed for this study. These items included “Do you have someone (colleagues, family, friends, etc.) who can talk or provide advice on work-related issues” for instrumental support and “Do you have someone (colleagues, family, friends, etc.) who supports or understands your attitudes toward work-related matters” for emotional support. Items were answered using a binary *yes*/*no* response format. The internal consistency coefficient for these items was 0.53.

**Work-related distress**

Work-related distress over the past month was assessed with six items. Respondents answered the extent to which they felt stressed in six aspects of work using a 5-point scale ranging from 1 (*I didn’t feel it at all*) to 5 (*I felt very strongly*). Respondents answer the items with the stem, “To what extent do you feel stressed about the following aspects of your work.” Items included “stress due to excessive work,” “stress due to work content,” “stress due to lack of decision making,” “stress due to unsatisfactory evaluation,” “not finding the work as worthy,” and “stress due to interpersonal relationships in the workplace.” Total scores were calculated by summing the scores of all items. The internal consistency coefficient for this measure was acceptable for this study, α = 0.79.

**Demographics**

We collected participants’ demographic information, including age, gender (women or men), marital status (married or single), years of experience working as a public health nurse, and the presence of lifetime traumatic experiences (yes or no).

### Procedures

Between July 27, 2015, and August 31, 2015, we distributed a paper-pencil version of the survey to all potential participants through the Japanese Nursing Association, the nursing associations in these four prefectures, and the Japanese Association of Public Health Nurses.

### Data analysis

We used the R version 4.0.5 for all statistical analyses [27]. As a variable-centered approach, we conducted hierarchical multiple regression analysis for STS total scores as a dependent variable using the R package psych [28]. In the first step, region, gender, marital status, lifetime traumatic experiences, years of experience as a public health nurse, perceived support, and work-related distress were used as independent variables. In the second step, we entered the empathy subscales (i.e., empathic concern, personal distress, perspective taking, and fantasy) as independent variables. We computed bootstrap confidence intervals for the coefficients of the second step with 1,000 bootstrap samples (percentile bootstrap method) using the R package boot [29, 30].

Second, as a person-centered approach, we conducted a latent profile analysis using a Gaussian finite mixture model for scores of empathic concern, personal distress, perspective taking, fantasy, and STS using the R package mclust [31]. To confirm the number of groups resulting from the latent profile analysis, we used the bootstrap method with 999 bootstrap samples (nonparametric bootstrap method). The variables in the model were standardized. We used the Bayesian information criterion (BIC) to compare models with different numbers of groups. The model with the largest BIC indicates the optimal model.

Additionally, we conducted a series of one-way ANOVAs to compare scores of each subscale of empathy, burnout, and STS between the groups classified by the latent profile analysis. We conducted chi-square tests for the difference in the categorical variables among the categorized groups using the R packages RVAidememoire [32]. (See the supplemental materials for the R codes used in the analyses).

### Missing data

After excluding the respondents who did not complete any items on the STSS-J, IRI-J, or MBI-J, 1,798 responses (2.38%) had missing data. We imputed these missing data with a random forest imputation algorithm using the R package missForest [33, 34].

## Results

### Demographics and correlations among the study variables

The participants (97.2% women) worked in the Tohoku (69.0%) or Saitama (31.0%) regions. Approximately 70% were married (68.1%). Most reported no lifetime traumatic experiences before the 2011 Great East Japan Earthquake (77.8%). The number of years of experience as a public health nurse varied (range: 5 to ≥ 30 years; Table 1).

**Table 1:** Demographic characteristics of the tohoku and saitama samples

		Tohoku (n = 694)		Saitama (n = 312)		Total	
		*n*	%	*n*	%	*n*	%
Sex							
	Men	20	2.9	8	2.6	28	2.8
	Women	674	97.1	304	97.4	978	97.2
Marital status							
	Married	482	69.5	203	65.1	685	68.1
	Not married	212	30.5	109	34.9	321	31.9
Traumatic exp.							
	Yes	123	17.7	100	32.1	223	22.2
Career (years)							
	≤ 4	141	20.3	65	20.8	206	20.5
	5–9	77	11.1	46	14.7	12	12.2
	10–14	72	10.4	55	17.6	127	12.6
	15–19	97	14.0	56	17.9	153	15.2
	20–24	86	12.4	39	12.5	125	12.4
	25–29	91	13.1	33	10.6	124	12.3
	≥ 30	130	18.7	18	5.8	148	14.7
		Mean	SD	Mean	SD	Mean	SD
Perceived support		3.97	0.18	3.96	0.25	3.97	0.21
Work-related distress		13.33	4.45	13.82	3.97	13.48	4.31
Empathy							
	Empathic concern	25.98	3.29	25.44	3.16	25.81	3.26
	Personal distress	21.63	3.87	21.92	4.03	21.72	3.92
	Perspective taking	23.90	3.64	24.20	3.43	23.99	3.58
	Fantasy	19.44	5.08	19.80	5.08	19.55	5.08
Burnout							
	Exhaustion	20.06	7.22	21.26	7.38	20.43	7.29
	Cynicism	13.94	6.27	14.66	6.90	14.16	6.48
	Professional efficacy	17.71	6.36	17.95	6.73	17.79	6.47
STS		27.45	10.51	32.07	12.40	28.88	11.33

Note. Traumatic exp. = lifetime traumatic experience; SD = standard deviation; STS = secondary traumatic stress

Pearson’s correlation coefficients between the study variables had effect sizes ranging from small to large (Table 2). We classified the Pearson correlation coefficients in small, medium, or large effect sizes based on the criteria (small = 0.10, medium = 0.30, large = 0.50) proposed by Cohen [35]. Empathic concern and perspective taking had a medium effect size, and empathic concern and other empathy subscales had small effect sizes. Furthermore, personal distress and exhaustion had a medium effect size, but all other empathy and burnout subscales had small effect sizes. STS had a medium effect size with personal distress, but small effect sizes with the other empathy subscales. Furthermore, STS had medium effect sizes with exhaustion and cynicism, and a small effect size with professional efficacy. Finally, exhaustion and cynicism had a significant effect size, but the relationships among other burnout subscales had small effect sizes.

**Table 2:** Correlation (pearson correlation coefficients) matrix, means, and standard deviations for the study variables

	1. EC	2. PT	3. PD	4. FS	5. STS	6. Ex	7. Cy	8. PE
1		0.31	0.16	0.21	0.01	0.03	− 0.12	0.11
2			− 0.07	0.05	− 0.04	− 0.03	− 0.11	0.11
3				0.37	0.35	0.35	0.29	− 0.31
4					0.17	0.14	0.06	0.01
5						0.42	0.40	− 0.10
6							0.66	− 0.07
7								− 0.18

*Note*. EC = empathic concern (empathy); PT = perspective taking (empathy); PD = personal distress (empathy); FS = fantasy (empathy); STS = secondary traumatic stress; Ex = exhaustion (burnout); Cy = cynicism (burnout); PE = professional efficacy (burnout)

### Hierarchical multiple regression predicting STS

We conducted a hierarchical multiple regression to examine the association between dispositional empathy and STS. We found no multicollinearity issues between the independent variables (variance inflation factor range: 1.05–1.40). Results showed that the model improved from Step 1 (R^2^_adj_ = 0.19) to Step 2 (R^2^_adj_ = 0.26), *F*(4, 73.60) = 24.81, *p* < .001. In Step 2, working in Saitama prefecture, lifetime traumatic experiences and work-related, and personal distress were associated with higher STS scores. Emotional support was related to lower STS scores (Table 3).

**Table 3:** Hierarchical multiple regression analysis for secondary traumatic stress as a dependent variable

	B	β	SE (β)	95% CI (Lower)	95% CI (Higher)
Intercept	18.31*	0.02	0.17	− 0.29	0.33
Region (Saitama) vs. Tohoku	3.40**	0.30	0.06	0.17	0.43
Gender (women) vs. men	-2.26	− 0.20	0.17	− 0.52	0.12
Marital status (yes) vs. no	0.08	0.01	0.07	− 0.13	0.15
Traumatic experiences (yes) vs. no	3.84**	0.34	0.07	0.19	0.49
Career (years)	0.04	0.01	0.03	− 0.06	0.07
Perceived support	-3.44*	− 0.06	0.03	− 0.15	0.04
Work-related distress	0.71**	0.27	0.03	0.21	0.33
Empathic concern (empathy)	-0.11	− 0.03	0.03	− 0.09	0.03
Perspective taking (empathy)	-0.05	− 0.01	0.03	− 0.07	0.05
Personal distress (empathy)	0.78**	0.27	0.03	0.20	0.34
Fantasy (empathy)	0.09	0.04	0.03	− 0.02	0.10

*Note.* Region compares Tohoku with Saitama as the baseline. STS = secondary traumatic stress; B = unstandardized coefficient; β = standardized coefficient; SE = standard error; 95% CI = 95% bootstrap confidence intervals; IRI = interpersonal reactivity index. ** p < .001; * *p* < .05

### Latent profile analysis for dispositional empathy and STS

**Fig. 2:**
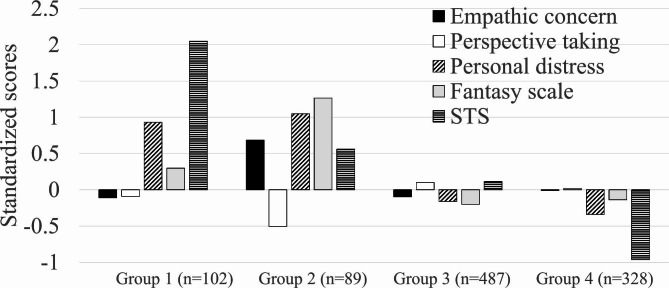
Standardized scores of the study variables classified by the groups identified in the latent profile analysis. Note. STS = secondary traumatic stress

We conducted a latent profile analysis to explore the sample classification based on the IRI subscales and total STSS scores. Results suggested the model with four groups as optimal based on BIC (-13647.85; model estimation = EVE). Other classifications with different numbers of groups yielded smaller BIC values (1 group = -13945.44, 2 groups = -13814.27, 3 groups = -13873.53, 5 groups = could not be calculated). These four groups comprised Group 1 (*n* = 102, 10.1%), Group 2 (*n* = 89, 8.8%), Group 3 (*n* = 487, 48.4%), and Group 4 (*n* = 328, 32.6%; Fig. 2). Group 1 had the highest STS scores and high personal distress scores. Group 2 represented participants with the highest empathic concern, personal distress, and fantasy scores, along with the second-highest STS level. A moderate STS level characterized Group 3. Finally, Group 4 had the lowest STS and personal distress scores.

### Differences between groups

We conducted a series of one-way ANOVAs and chi-square tests to compare the study variables between the groups identified in the latent profile analysis. We used the Benjamini-Hochberg procedure to adjust the false discovery rate for the follow-up tests [36]. The Benjamini-Hochberg procedure is a powerful approach to decreasing false positives. All variables significantly differed between the groups, F range = 7.27–1264.3, all *p*s < 0.001 (Table 4). The frequencies of regions were significantly different among the groups, χ^2^(3) = 32.96, *p* < .001. The post-hoc follow-up test showed a significant difference between Group 1 and Group 3 (adjusted *p* < .001) and between Group 1 and Group 4 (adjusted *p* < .001). There was a difference in lifetime traumatic experiences depending on the groups, χ^2^(3) = 47.2, *p* < .001. Results of the posthoc comparisons showed a significant difference between Group 1 and Group 2 (*p* = .03), Group 1 and Group 3 (*p* < .001), Group 1 and Group 4 (*p* < .001), and Group 2 and Group 4 (*p* = .039). We ran a Fisher’s exact test for gender and the group because two cells had fewer than 5 participants. Results of a Fisher’s exact test showed that gender was not different among the groups, *p* = .647.

**Table 4:** Results of the comparisons among the groups identified in the latent profile analysis

		Group 1 (n = 102, 10.1%)	Group 2 (*n* = 89, 8.8%)	Group 3 (n = 487, 48.4%)	Group 4 (*n* = 328, 32.6%)		
		Mean				*F*	Significant posthoc comparisons
EC (empathy)		25.45	28.04	25.50	25.78	22.40*	1 < 2, 2 > 3, 2 > 4
PT (empathy)		23.67	22.18	24.35	24.05	8.38*	1 > 2, 2 < 3, 2 < 4
PD (empathy)		25.37	25.83	21.10	20.39	109.68*	1 > 3, 1 > 4, 2 > 3, 2 > 4, 3 > 4
FS (empathy)		21.07	26.00	18.53	18.85	118.14*	1 < 2, 1 > 3, 1 > 4, 2 > 3, 2 > 4
Ex		27.55	23.61	20.27	17.60	68.83*	All
Cy		20.79	16.03	13.84	12.07	44.27*	All
PE		16.36	15.84	18.00	18.44	7.27*	1 < 3, 1 < 4, 2 < 3, 2 < 4
STS		52.12	35.24	30.20	17.98	1264.3*	All
		Frequency (%)				χ^2^	Significant posthoc comparisons
Region						32.96*	1 vs. 2, 1 vs. 3, 1 vs. 4, 2 vs. 4, 3 vs. 4
	Tohoku	48 (47.1)	58 (65.2)	336 (69.0)	252 (76.8)		
	Saitama	54 (52.9)	31 (34.8)	151 (31.0)	76 (23.2)		
Marital status						7.16	
	Married	59 (57.8)	61 (68.5)	346 (71.0)	219 (66.8)		
	Not married	43 (42.2)	28 (31.5)	141 (29.0)	109 (33.2)		
Traumatic exp.						47.2*	1 vs. 2, 1 vs. 3, 1 vs. 4, 2 vs. 4
	Yes	48 (47.1)	25 (28.1)	98 (20.1)	52 (15.9)		
	No	54 (52.9)	64 (71.9)	389 (79.9)	276 (84.1)		
Gender						p-value for Fisher’s exact test	
	Men	2 (2.0)	1 (1.1)	17 (3.5)	8 (2.4)	0.647	
	Women	100 (98.0)	88 (98.9)	470 (96.5)	320 (97.6)		

*Note*. EC = empathic concern; PD = personal distress; PT = perspective taking; FS = fantasy; Ex = exhaustion; Cy = cynicism; PE = professional efficacy; STS = secondary traumatic stress; Traumatic exp. = lifetime traumatic experiences. * p < .001

## Discussion

The present study explored the psychological distress among a sample of Japanese public health nurses, and its association with dispositional empathy. First, we examined variables that could have been associated with STS using a variable-centered approach. The results showed that STS was associated with personal distress, lifetime traumatic experiences, and work-related stress. Additionally, higher perceived support scores were related to lower STS scores, consistent with previous findings [37]. Higher personal distress, a self-oriented emotional component of dispositional empathy, was related to higher STS in physicians [16]. Personal distress indicates sensitivity to another person’s negative affect. Healthcare professionals with high personal distress may perceive someone’s negative affect as their own, facilitating the development of STS.

Even though personal distress does not always lead to STS, it contributes to the development of STS in several ways [38]. One such model has included personal distress as a mediator [39]. In this model, stressful situations facilitated personal distress that would, in turn, exacerbate the development of STS. This model suggests that people experience distress when exposed to another’s suffering. This heightened distress might trigger the development of STS.

Another possibility is that empathy and STS have a nonlinear relationship. There might be an empathy threshold where empathy affects STS more strongly. This model is often depicted as a cusp catastrophe model [40]. Some empirical evidence suggests post-traumatic stress symptoms suddenly shift from a lower level to an elevated level based on the threshold of coping self-efficacy over time [41]. Another study reported that cumulative stress and trauma contribute to the development of thought disorders (depicted as non-symptomatic vs. symptomatic). The previous findings suggest that such a sudden shift in behaviors might be a common phenomenon in post-trauma.

Furthermore, coping strategies can affect the relationship between dispositional empathy and STS. For example, higher empathy is related to lower avoidant coping (e.g., shifting responsibilities, abandonment), which is further related to lower psychological distress among Japanese workers [42]. When empathy is elevated, individuals tend to engage in cognitive reappraisal that is, in turn, related to lower psychological distress. Thus, people might have low STS levels when engaging in active coping strategies even with elevated personal distress.

Our person-centered approach found that our sample of public health nurses could be classified into four groups based on dispositional empathy and STS levels. Group 1 had the highest STS level, with higher personal distress than Groups 3 and 4. This group reflects the findings of the variable-centered approach, demonstrating the association between high personal distress levels and high STS levels. Compared to Group 1, Group 2 presented moderate STS, a lower level of perspective-taking, higher levels of empathic concern and fantasy, and almost equivalent but the highest level of personal distress. Based on previous studies, personal distress has consistently been negatively associated with mental well-being, such as compassion satisfaction, and positively associated with negative psychological responses, such as STS and burnout [16, 18, 43]. Interestingly, empathic concern and perspective-taking are significantly associated with positive and negative psychological responses (compassion satisfaction and STS), suggesting that human empathy is a double-edged blade. Given these prior findings, the fact that the STS of Group 2 is smaller than that of Group 1 is unexpected, but the relationship between empathic characteristics and psychological responses is complex. Group 2, those with the highest empathic concern, includes emotionally reactive individuals who respond to people in need with compassion and concern. They might have high levels of compassion satisfaction, which might have acted as a powerful confounding factor that mitigates STS. However, these are only speculations and need to be empirically tested in future research.

Elevated empathic concern, personal distress, and fantasy can influence a positive psychological response to help others. Interestingly, in addition to Groups 1 and 2 having relatively lower professional efficacy, they had the highest personal distress. Moreover, Group 2 also had the highest empathic concern, characterized by highly identifying their feelings with others’ sufferings. These characteristics might hinder their ability to have positive attitudes toward their work (i.e., professional efficacy). These findings suggest the nuanced nature of dispositional empathy concerning the consequences of caring.

Further examinations revealed that Group 3 had higher personal distress and burnout (i.e., exhaustion, cynicism) than Group 4. Burnout has the same patterns as STS, with Group 1 having the highest burnout and Group 4 having the lowest. These findings are consistent with other findings that burnout can be a vital precursor to future STS [37].

In comparing the presence of lifetime traumatic experiences between the groups, the frequency was higher in the order of Groups 1 to 4. This trend might contribute to the lower STS levels in Group 2 than in Group 1, even though they have equivalent levels of personal distress. These findings indicate that participants with lifetime traumatic experiences are more likely to be classified into groups with relatively high STS and personal distress. Consistent with these findings, a meta-analysis showed that STS and personal traumatic history had a small but positive effect size (r = .19) in professionals working with people suffering from trauma [44]. The present study adds to the existing literature, finding that people with elevated STS exhibit increased personal distress.

This study included an unexpected finding in the relationship between the region and STS. The finding indicates that public health nurses in Saitama had higher STS than those in the Tohoku region where the 2011 Great East Japan Earthquake occurred. Although speculative, this unexpected finding might be due to two reasons. Many public health nurses from other regions (12,000, the highest number ever to support people in the disaster area) went to the Tohoku region to support the public health nurses working there. As such, they might have enhanced perceived support, reducing STS. Additionally, Saitama is in the Tokyo metropolitan area and has fewer public health nurses per population than the Japanese national average [45]. Due to these regional characteristics, public health nurses in Saitama might have been exposed to indirect traumatic stress more frequently than those in the Tohoku region, which might contribute to elevated STS among those in Saitama.

### Limitations

Despite demonstrating that classifying public health nurses based on dispositional empathy and STS provides new insights into the role of dispositional empathy, this study has several limitations. First, this is a cross-sectional study. We could not test the effect of dispositional empathy in classifying public health nurses over time. Future studies must investigate how STS or burnout changes across these four groups over time. Furthermore, our study provides potentially useful information on different classes based on dispositional empathy and STS. These findings should be replicated in other populations of nurses or other healthcare professionals. As latent profile analysis is sensitive to slight changes in the data, different occupations might have distinct classifications.

## Conclusions

The present study examined the relationship between dispositional empathy and STS in the variable-centered, including person-centered approaches. Our findings suggested four groups of public health nurses based on dispositional empathy and STS in the person-centered approach, which enables us to identify nuances among the groups that the variable-centered approach could not. In addition, we demonstrated that personal distress is associated with STS in the variable-centered approach. Despite its several limitations, this study provides new insights into the roles of dispositional empathy in Japanese public health nurses. Future studies must replicate our findings and investigate more differences between the classes we identified in this study.

### Electronic supplementary material

Below is the link to the electronic supplementary material.

Supplemental Material. R codes for the data cleaning, data processing, and data analysis.

## Data Availability

The datasets used and analyzed during the present study are available from the corresponding author upon reasonable request.

## List of abbreviations

BIC: Bayesian Information Criterion
Cy: Cynicism
EC: Empathic concern
Ex: Exhaustion
FS: Fantasy
IRI-J: Interpersonal Reactivity Index-Japanese version
MBI-J: Maslach Burnout Inventory-Japanese version
PD: Personal distress
PE: Professional efficacy
PT: Perspective taking
SD: Standard deviation
STS: Secondary traumatic stress
STSS-J: Secondary Traumatic Stress Scale-Japanese version
