# Supplementary material for: Japanese public health nurses classified based on empathy and secondary traumatic stress: variable-centered and person-centered approaches
Source: BMC Psychiatry. 2023 Oct 2;23:710. doi: 10.1186/s12888-023-05198-6 (PMC10544614; doi:10.1186/s12888-023-05198-6)
Supplement: Supplementary file 1 — Supplemental Material. R codes for the data cleaning, data processing, and data analysis. [file 12888_2023_5198_MOESM1_ESM.docx]

**Supplemental Material. R Codes for the Data Cleaning, Data Processing, and Data Analysis**

load("IRI.RData")

library(dplyr)

#========== hierarchical multiple regression. ============================

# step1: demographics and environment working variable

hlmmod1 <- lm(STSS_totaln ~ tohoku_saitama+sex+marriage+trauma+careern+supportn+workstressn,data = IRI)

summary(hlmmod1)

# step2: IRI

hlmmod2 <- lm(STSS_totaln ~ tohoku_saitama+sex+marriage+trauma+careern+supportn+workstressn+ECn+PDn+PTn+FSn,data = IRI)

summary(hlmmod2)

anova(hlmmod1,hlmmod2)

# step2: unstandardized coefficients

hlmmod3 <- lm(STSS_total ~ tohoku_saitama+sex+marriage+trauma+career+support+workstress+EC+PD+PT+FS,data = IRI)

summary(hlmmod3)

# check the collinearity

library(car)

vif(hlmmod4)

# bootstrap

library(boot)

boot.fn = function(data, index) + return(coef(lm(STSS_totaln ~tohoku_saitama+sex+marriage+trauma+careern+supportn+workstressn+ECn+PDn+PTn+FSn,data = IRI, subset=index)))

set.seed(2020)

boot_reg <- boot(IRI,boot.fn,1000)

boot.ci(boot_reg,index = 1,type = "perc")

boot.ci(boot_reg,index = 2,type = "perc")

boot.ci(boot_reg,index = 3,type = "perc")

boot.ci(boot_reg,index = 4,type = "perc")

boot.ci(boot_reg,index = 5,type = "perc")

boot.ci(boot_reg,index = 6,type = "perc")

boot.ci(boot_reg,index = 7,type = "perc")

boot.ci(boot_reg,index = 8,type = "perc")

boot.ci(boot_reg,index = 9,type = "perc")

boot.ci(boot_reg,index = 10,type = "perc")

boot.ci(boot_reg,index = 11,type = "perc")

boot.ci(boot_reg,index = 12,type = "perc")

#========== model based clustering. ============================

library(mclust)

IRI_mclust <- IRI %>% dplyr::select(ECn,PDn,PTn,FSn,STSS_totaln)

mod <- Mclust(IRI_mclust)

summary(mod)

mclustmod1 <- Mclust(IRI_mclust, G = 4, modelName = "EVE")

summary(mclustmod1)

class <- mclustmod1$classification

mclustresult <- cbind(IRI,class)

# ordered group No (#group4→2, group2→3, group3→4)

mclustresult$class <- ifelse(mclustresult$class==1,"group1",ifelse(mclustresult$class==2,"group3", ifelse(mclustresult$class==3,"group4",ifelse(mclustresult$class==4,"group2", mclustresult$class))))

# bootstrap

boot <- MclustBootstrap(mclustmod1,nboot = 999,type = "bs")

plot(boot,what = "pro")

# one-way ANOVA

library(tableone)

# EC

oneway.test(EC~class,data = mclustresult)

tapply(mclustresult$EC,mclustresult$class,mean)

pairwise.t.test(mclustresult$EC,mclustresult$class,p.adjust.method = "BH")

# PD

oneway.test(PD~class,data = mclustresult)

tapply(mclustresult$PD,mclustresult$class,mean)

pairwise.t.test(mclustresult$PD,mclustresult$class,p.adjust.method = "BH")

# PT

oneway.test(PT~class,data = mclustresult)

tapply(mclustresult$PT,mclustresult$class,mean)

pairwise.t.test(mclustresult$PT,mclustresult$class,p.adjust.method = "BH")

# FS

oneway.test(FS~class,data = mclustresult)

tapply(mclustresult$FS,mclustresult$class,mean)

pairwise.t.test(mclustresult$FS,mclustresult$class,p.adjust.method = "BH")

# MBI_Exhaustion

oneway.test(MBI_Exhaustion~class,data = mclustresult)

tapply(mclustresult$MBI_Exhaustion,mclustresult$class,mean)

pairwise.t.test(mclustresult$MBI_Exhaustion,mclustresult$class,p.adjust.method = "BH")

# MBI_Cynicism

oneway.test(MBI_Cynicism~class,data = mclustresult)

tapply(mclustresult$MBI_Cynicism,mclustresult$class,mean)

pairwise.t.test(mclustresult$MBI_Cynicism,mclustresult$class,p.adjust.method = "BH")

# MBI_ProfessionalEfficacy

oneway.test(MBI_ProfessionalEfficacy~class,data = mclustresult)

tapply(mclustresult$MBI_ProfessionalEfficacy,mclustresult$class,mean)

pairwise.t.test(mclustresult$MBI_ProfessionalEfficacy,mclustresult$class,p.adjust.method = "BH")

# STSS_total

oneway.test(STSS_total~class,data = mclustresult)

tapply(mclustresult$STSS_total,mclustresult$class,mean)

pairwise.t.test(mclustresult$STSS_total,mclustresult$class,p.adjust.method = "BH")

# demographic comparison

library(RVAideMemoire)

# region

chi <- with(mclustresult,table(tohoku_saitama,class))

chisq.test(chi)

fisher.multcomp(chi, p.method="BH")

# marital

chi <- with(mclustresult,table(marriage,class))

chisq.test(chi)

# post trauma event experience

chi <- with(mclustresult,table(trauma,class))

chisq.test(chi)

fisher.multcomp(chi, p.method="BH")

# career (for quality variable)

chi <- with(mclustresult,table(career,class))

expected <- chisq.test(chi)

expected$expected

fisher.multcomp(chi, p.method = "BH")

# gender (Fisher’s exact test)

chi <- with(mclustresult,table(sex,class))

fisher.test(chi)
